# Supplementary material for: Regulome analysis in B-acute lymphoblastic leukemia exposes Core Binding Factor addiction as a therapeutic vulnerability
Source: Nat Commun. 2022 Nov 21;13:7124. doi: 10.1038/s41467-022-34653-3 (PMC9678885; doi:10.1038/s41467-022-34653-3)
Supplement: Supplementary file 1 — Supplementary Information [file 41467_2022_34653_MOESM1_ESM.pdf]

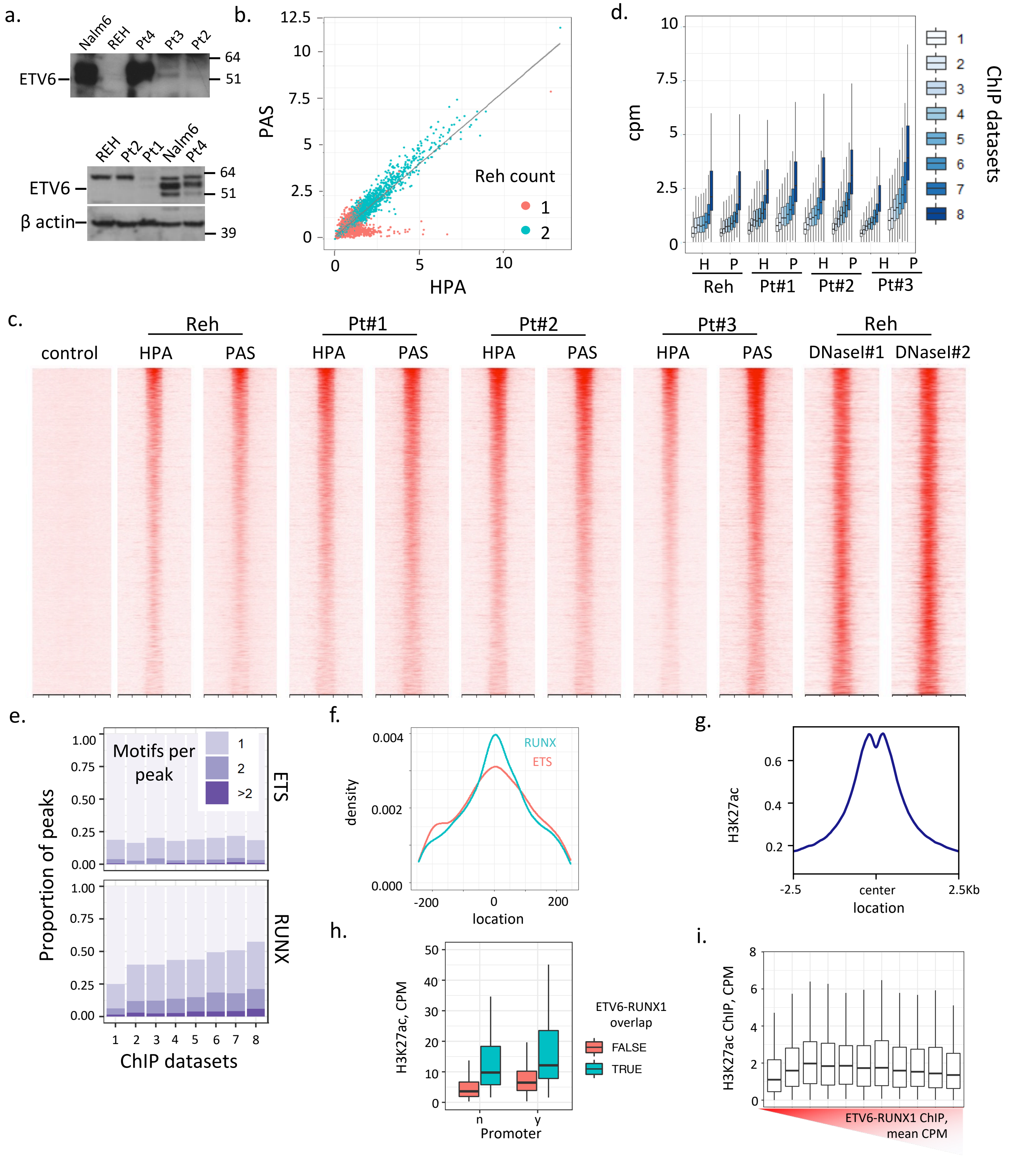

Supplementary Figure 1; related to Figure 1. Delineating the ETV6-RUNX1 regulome in childhood B-ALL
(a) Western blot for ETV6 in the indicated cell lines and patient (Pt) samples. Representative of two independent repeats. (b) Dotplot showing normalised read counts (counts per million, CPM) for a 500bp window centred on the summit for all ETV6-RUNX1 peaks identified for two independent ETV6 antibodies (HPA and PAS). Colours represent peaks called in one (1, red dot) or both (2, green dot) ChIP-seq datasets. (c) Heatmap showing ChIP-seq and DNaseI-hypersensitivity read density in a 3kb window centred on peak summits for all ETV6-RUNX1 peaks identified. (d) Boxplot showing distribution of normalised read counts for each of 8 ChIP-seq samples across all ETV6-RUNX1 peaks (n=6195) identified. Colour represents the number of ChIP datasets a peak was identified in. (e) Barplots showing the proportion of peaks, binned according to the number of ETV6-RUNX1 ChIP datasets in which they were identified, with the indicated number of RUNX/ETS motifs. (f) Density plot of RUNX and ETS motif distribution across a 500bp window centred on ETV6-RUNX1 ChIP peak summits. (g) H3K27ac ChIP-seq density for a 5kb window centred on the summit for the union of ETV6-RUNX1 peaks identified in REH and patient samples. (h) H3K27ac (CPM) at all H3K27ac peaks called by MACS (n=22198) subset by Promoter overlap and overlap with ETV6-RUNX1 peaks identified in all 8 datasets. (i) H3K27ac (CPM) for a 500bp window centred on the summit for the union of ETV6-RUNX1 peaks (n=6195), ordered by the mean ETV6-RUNX1 CPM and divided into 11 bins with equal numbers of peaks. Boxplots display median, inter-quartile range (box), minima and maxima (whiskers) (d, h, i). Source data are provided as a Source Data file.

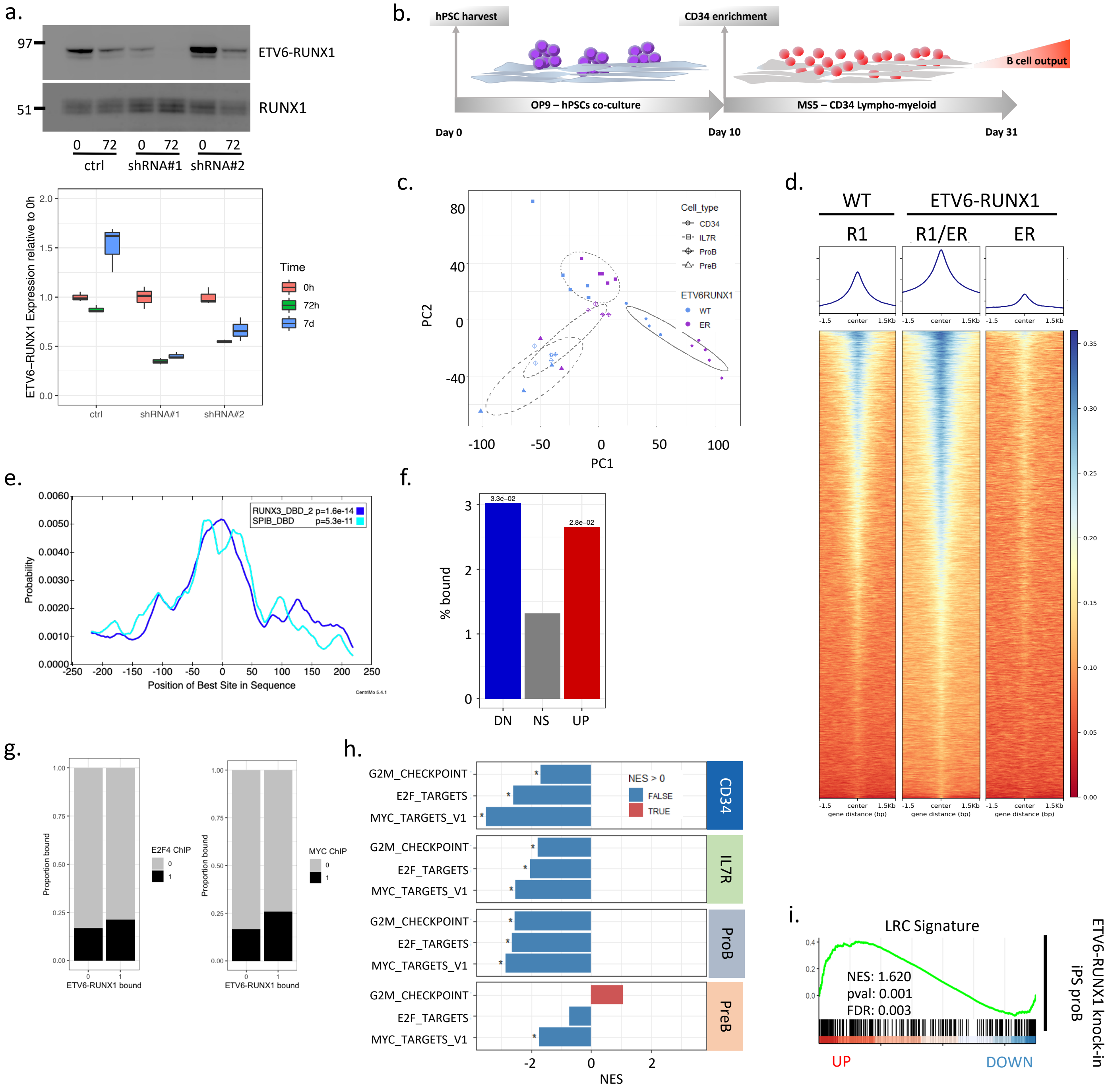

Supplementary Figure 2; related to Figure 2. *ETV6-RUNX1* induces transcriptional changes indicative of cell cycle repression in pre-leukemia

(a) Western blot and quantitative PCR showing ETV6-RUNX1 expression in Reh cells for vector control or ETV6-RUNX1 inducible shRNAs 0h, 72h or 7 days (7d) after addition of doxycycline (n=3). RUNX1 protein levels were unaffected by shRNAs targeting ETV6-RUNX1. (b) Schematic of hPSC B-cell differentiation protocol. (c) Principal component analysis of RNA-seq data from wild-type and ETV6-RUNX1 expressing iPSC-derived cell populations. (d) Heatmap showing normalized signal for ChIP-seq across 3kb regions centred on the summit of ETV6-RUNX1 binding sites identified in REH and patient samples. Chromatin from CD45+ haematopoietic cells differentiated from wild-type (WT) or ETV6-RUNX1-V5 knock-in IPSCs was immunoprecipitated with antibodies against RUNX1 or tagged ETV6-RUNX1 (V5) resulting in binding profiles for RUNX1 (R1), ETV6-RUNX1 (ER) or the two combined (R1/ER). (e) Density plot showing the distribution of the most highly enriched motifs identified by MEME for the union of RUNX1 and ETV6-RUNX1 peaks identified. (f) Barplot showing the percentage of genes identified as significantly different in ETV6-RUNX1-expressing proB cells that are associated with a RUNX1/ETV6-RUNX1 ChIP peak in CD45+ ChIP-seq. (g) Barplot showing the proportion of genes bound (1) or not (0) by ETV6-RUNX1 that are also bound by E2F4 or MYC. (h) Barplot showing normalized enrichment scores (NES) from GSEA for the indicated genesets against pre-ranked gene lists for ETV6-RUNX1 vs wild-type for each of the four populations indicated. * $p<0.05$. (i) GSEA for label retaining cell (LRC) signature genes against pre-ranked gene list for ETV6-RUNX1 vs wild-type in iPSC-derived proB cells. Boxplots display median, inter-quartile range (box), minima and maxima (whiskers) (a). One-way Fisher's exact test revealed significant over-representation of RUNX1/ETV6-RUNX1 bound genes in the indicated groups, Benjamini & Hochberg correction for multiple testing applied, only adjusted p-values <0.05 are shown (f). Pearson's Chi-squared test revealed a significant overlap between ETV6-RUNX1 and E2F4 (Chi-squared = 11.176, df = 1, p-value = 0.0008284), or MYC (Chi-squared = 45.488, df = 1, p-value = 1.536e-11) targets (g). Source data are provided as a Source Data file.

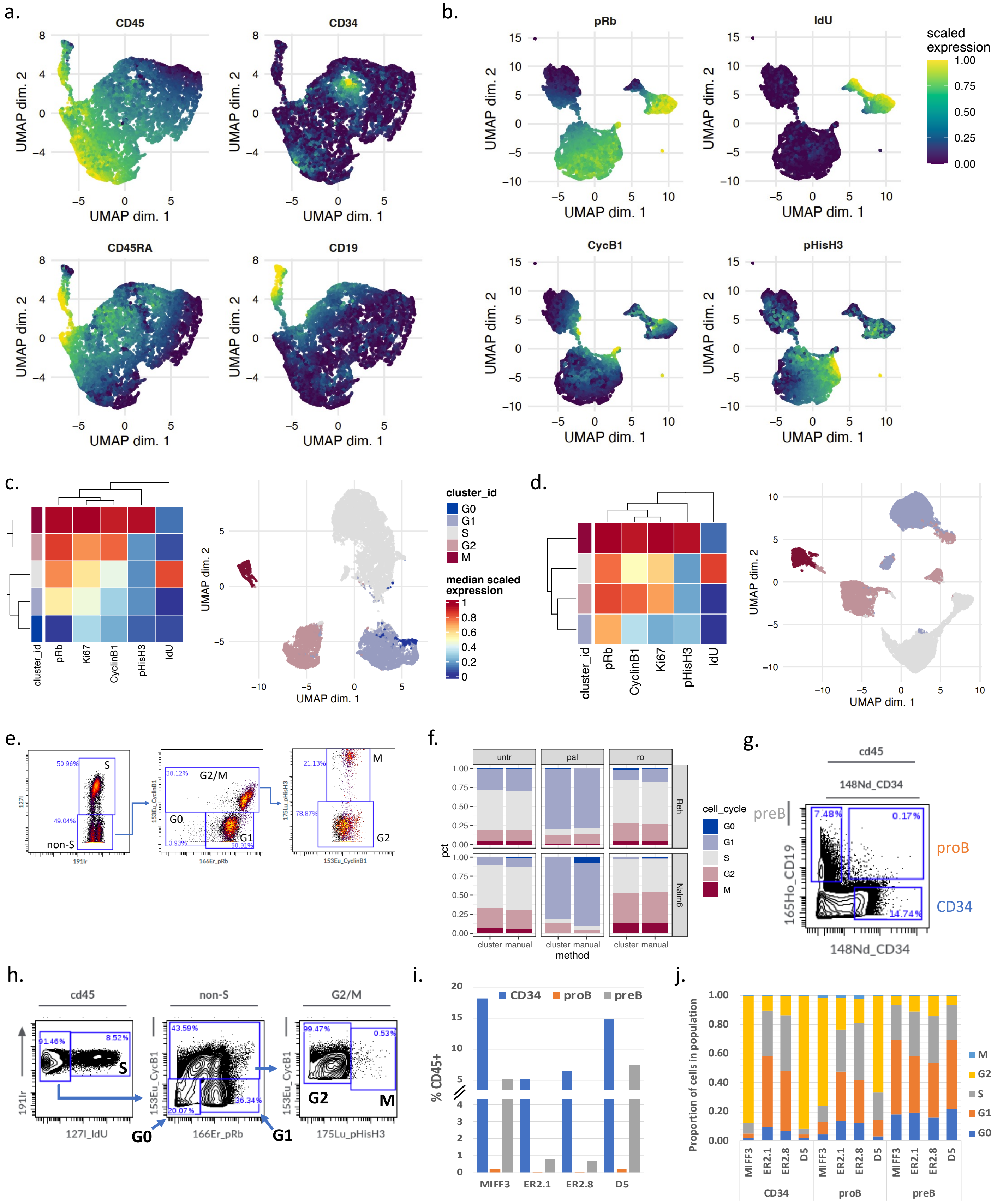

Supplementary Figure 3; related to Figure 3. Mass cytometry reveals an accumulation of *ETV6-RUNX1* expressing progenitor cells in S phase. (a-b). UMAP dimensionality reduction based on surface markers (a) or cell cycle markers (b) - colour represents scaled expression of the indicated markers - used to assign cell types and cell cycle phases for analysis in figure 3.(c,d) Clustering and UMAP dimensionality reduction for cell cycle markers pRb, Ki67, IdU, CycB1 and pHisH3 in Reh (c) and Nalm6 (d).(e) Illustrative manual gating to assign cell cycle phases based on the markers pRb, IdU, CycB1 and pHisH3. (f) Barplots showing cell cycle distribution of Reh and NALM-6 following treatment with CDK4/6 inhibitor Palbociclib (pal) or CDK1 inhibitor RO3306 (ro), quantified by clustering as in c/d or manual gating as in e. (i,j) Manual gating of CD34+ progenitors, proB and preB cells (i) and of cell cycle markers in IPSC differentiations. (k) Barplot showing percentage of CD45+ cells in the gates indicated in (i). (l) Proportion of cells in each of the cell cycle phases defined in (j) for each of the populations in (i). (k,l) MIFF3: (parental, 195473 cells); ER2.1/ER2.8 (ETV6-RUNX1 knock-in, 415891 and 379947 cells); D5 (reverted, 64072 cells). Source data are provided as a Source Data file.

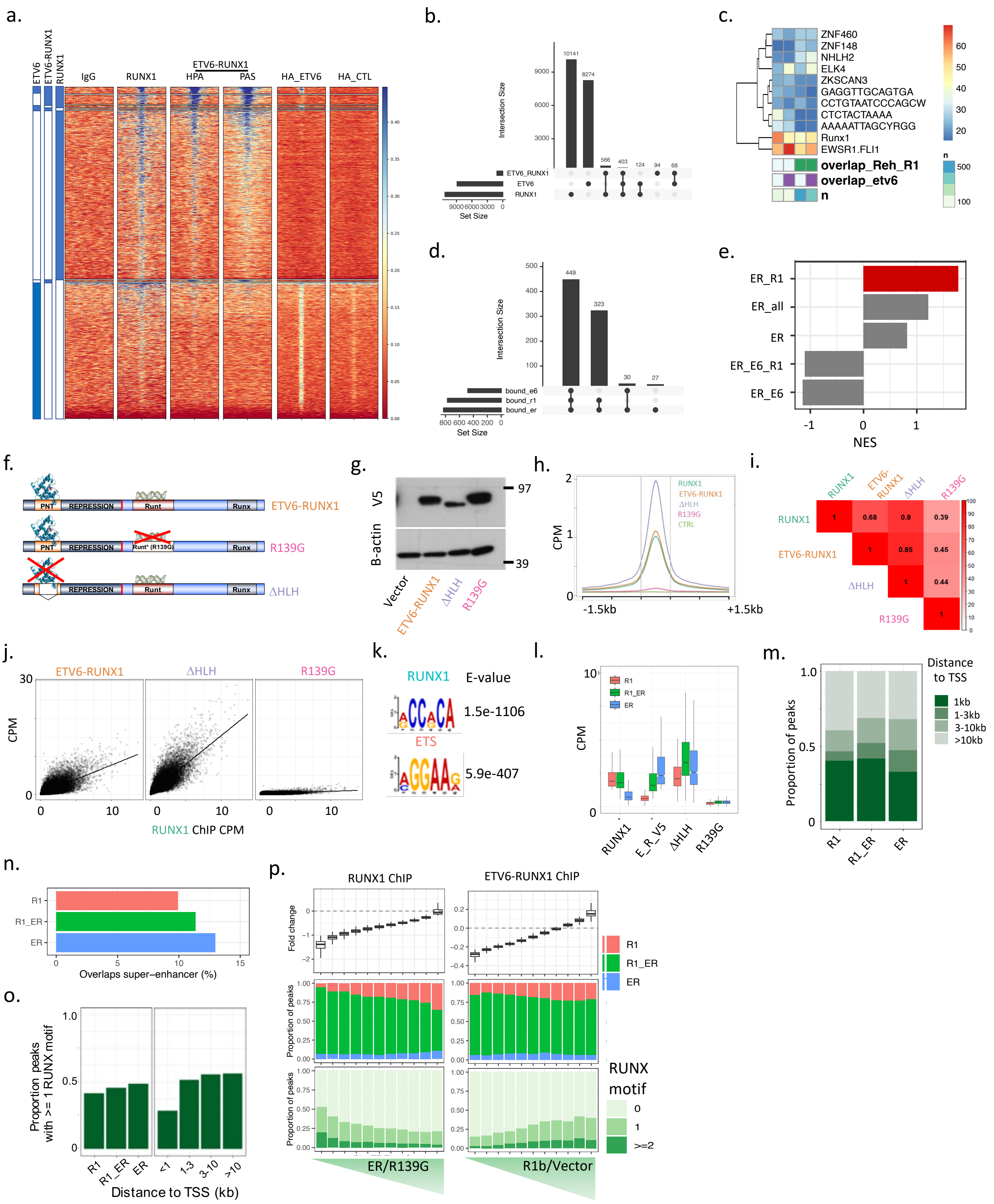

Supplementary Figure 4; related to Figure 4. ETV6-RUNX1 binds DNA through the Runt domain, competing with native RUNX1 (a) Heatmap showing ChIP signal for the indicated IPs in REH. Peaks were grouped according to the overlap between RUNX1, ETV6-RUNX1 and ETV6 ChIP, as shown in (b). (c) Heatmap showing the percentage of ETV6-RUNX1 peaks, subdivided according to their overlap with RUNX1 and ETV6, containing the indicated motifs. (d) Barplot showing the intersection of genes associated with one or more peak from the indicated ChIPs. (e) Gene set enrichment analysis for the genesets from (D) against a list of genes ranked by response to ETV6-RUNX1 knock-down. Red indicates padj<0.05. (d,e) er: ETV6-RUNX1; r1: RUNX1; e6: ETV6; er_all: all ETV6-RUNX1 peaks. (f) Schematic of ETV6-RUNX1 and R139G/ΔHLH mutants. R139G (runt domain point mutation); PNT (pointed domain, required for multimerization). (g) Western blot showing ETV6-RUNX1 mutants (detected with anti-V5) and B-actin loading control. Representative of two independent repeats. (h) Average profile plot of ChIP-seq signal across all RUNX1/ETV6-RUNX1 peaks identified in a 3kb window centred on peak summits. See next page.

Supplementary Figure 4; related to Figure 4. ETV6-RUNX1 binds DNA through the Runt domain, competing with native RUNX1

(i) Correlation matrix for ChIP (datasets based on normalised read counts (counts per million) for a 500bp window centred on the peak summit across all RUNX1/ETV6-RUNX1 peaks identified. (j) Dotplots of normalised read counts (counts per million (CPM)) for RUNX1 vs each ETV6-RUNX1 dataset. (k) MEME enrichment of motifs in sites bound by RUNX1 and/or ETV6-RUNX1. (l) Boxplot of CPM for peaks (n=19092) classified in (Figure 4c) for RUNX1 or ETV6-RUNX1 ChIP datasets. (m) Proportion of peaks in the indicated bins relative to TSS for each of the peak sets classified in Figure 4c. (n) Percentage of peaks, classified as in Figure 4c, overlapping with a super-enhancer. (o) Proportion of peaks with >= 1 RUNX1 motif mapped to a 500bp window centred on the peak summits for peaks binned according to classifications in Figure 4c (left panel) or distance from the TSS (right panel). (p) Differences in FLAG-RUNX1b (ETV6-RUNX1 vs R139G) or V5-ETV6-RUNX1 (RUNX1 vs Vector) were calculated using DiffBind and peaks (n=34867) ranked from the most significantly down- to most significantly up-regulated (see Figure 4e) and divided into 11 bins containing equal numbers of peaks. Boxplots of fold changes (top) and barplots of peaks distribution for classifications in Figure 4c (middle) and RUNX motif counts (bottom) are shown. Boxplots display median, inter-quartile range (box), minima and maxima (whiskers) (l,p), notches indicate 95% confidence intervals (l). Source data are provided as a Source Data file.

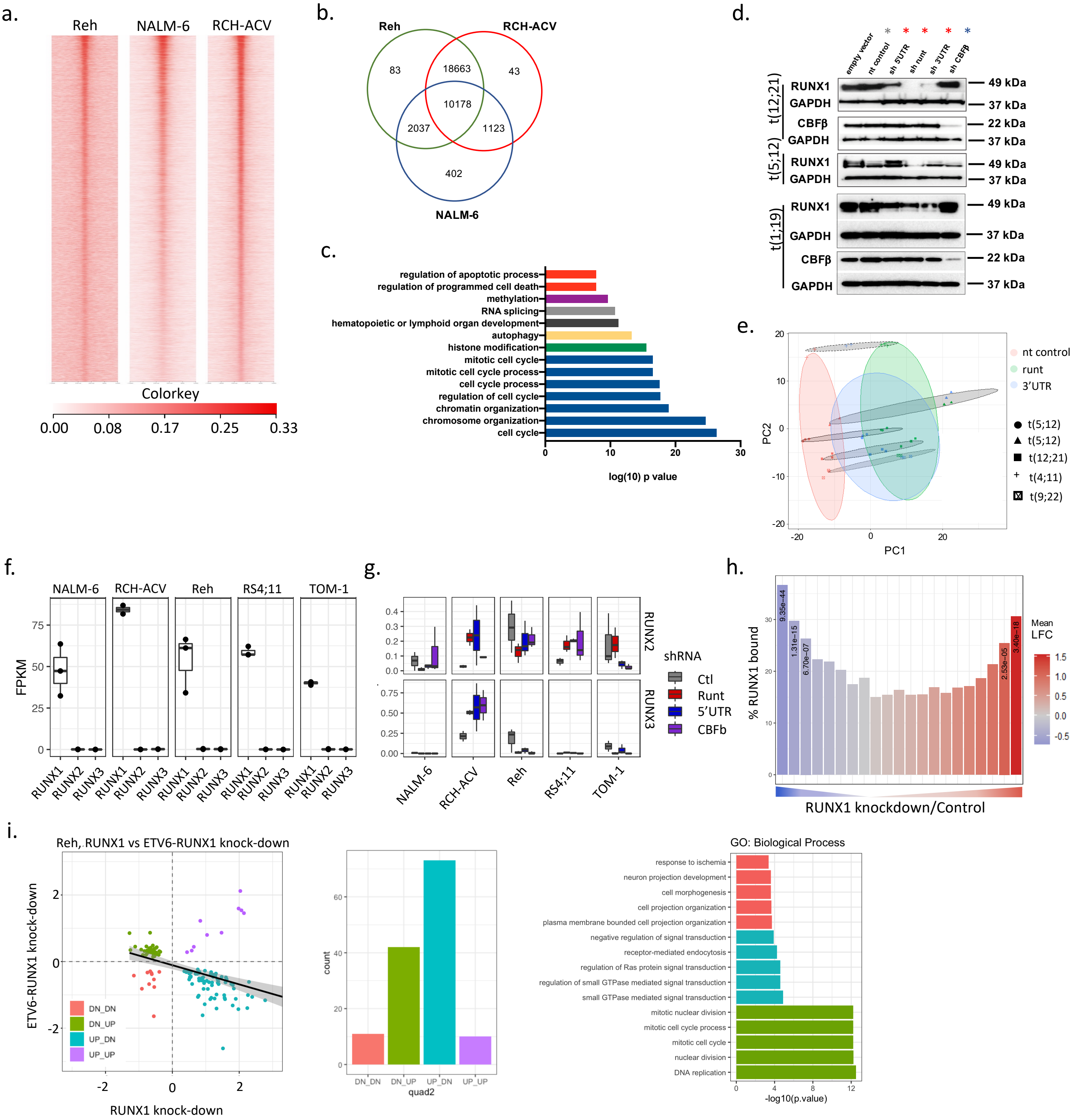

Supplementary Figure 5; related to Figure 5. Defining a core RUNX1 program in B-ALL reveals antagonism between ETV6-RUNX1 and native RUNX1 in cell cycle regulation

(a) Heatmaps showing RUNX1 ChIP-seq signal across all identified RUNX1 binding sites for the indicated cell lines in a 3kb window centred on peak summits. (b) Venn diagram showing overlap of RUNX1 ChIP peaks from Reh, RCH-ACV and NALM-6. (c) Bar plot showing significantly enriched biological processes from GO term analysis (selected biological processes shown). (d) Western blots of Reh, NALM-6 and RCH-ACV cells transduced with control (empty and non-targeting), RUNX1 or CBFb shRNAs. Protein levels are measured 48h after transduction. Asterisks indicate control (grey), RUNX1 (red) and CBFβ (blue) shRNAs used in subsequent experiments. (e) Dot plot of principal components 1 and 2 showing RUNX1 shRNA and control samples for 5 cell lines. Ellipses show 80% confidence intervals for the indicated groupings. (f,g) Boxplot of FPKM values for RUNX1, 2 and 3, in the indicated cell lines (f) and following RUNX1/CBFβ knock-down (g) n=3 biologically independent samples. (h) Bar plot showing percentage of genes bound by RUNX1 for genes binned according to response to RUNX1 knockdown. LFC: Log2 Fold Change. (i) Comparison of differentially expressed genes in Reh response to RUNX1 and ETV6-RUNX1 knock-down. Left: dotplot of log2 fold changes. Middle, barplot showing number of genes in each quadrant, indicated by colour. Right: GO-term analysis for genes in each quadrant. The 5 most significant Biological Processes for each quadrant are shown. Boxplots display median, inter-quartile range (box), minima and maxima (whiskers) (f,g). One-way Fisher's exact test revealed significant over-representation of RUNX1 bound genes in the indicated groups, Benjamini & Hochberg correction for multiple testing applied, only adjusted p-values <0.05 are shown (h). Source data are provided as a Source Data file.

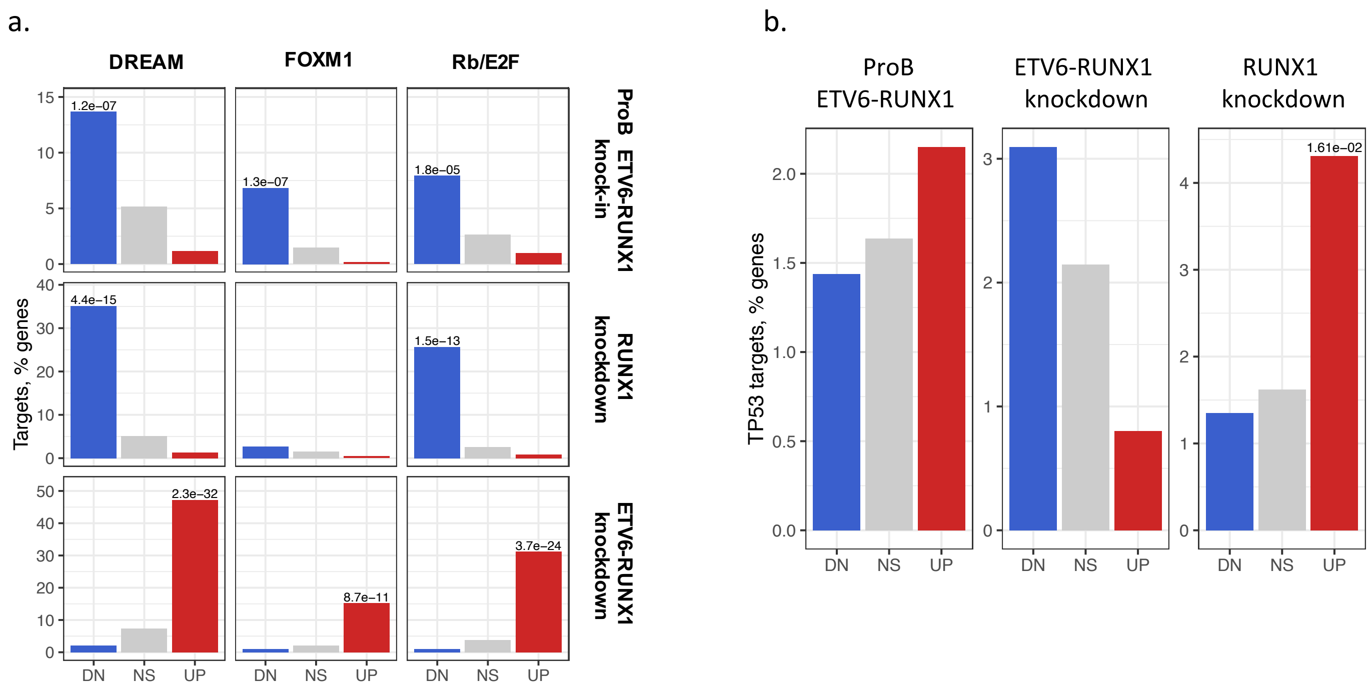

Supplementary Figure 6; related to Figure 6. A balance between CBF complex and ETV6-RUNX1 on the P53 regulated cell cycle-apoptosis axis promotes a silent pre-leukemic state

(a,b) Bar plots showing the percentage of genes significantly up- (UP), or down- (DN) regulated or not significant (NS) in ETV6-RUNX1 knock-in iPSC-derived proB cells or following ETV6-RUNX1 or RUNX1 knockdown which were defined as DREAM, Rb/E2F, or FOXM1 targets (a) or direct TP53 targets (Fischer et al., 2016) (b). One-way Fisher's exact test revealed significant over-representation of differentially expressed genes in the indicated groups, Benjamini & Hochberg correction for multiple testing applied, only adjusted p-values <0.05 are shown (a,b). Source data are provided as a Source Data file.

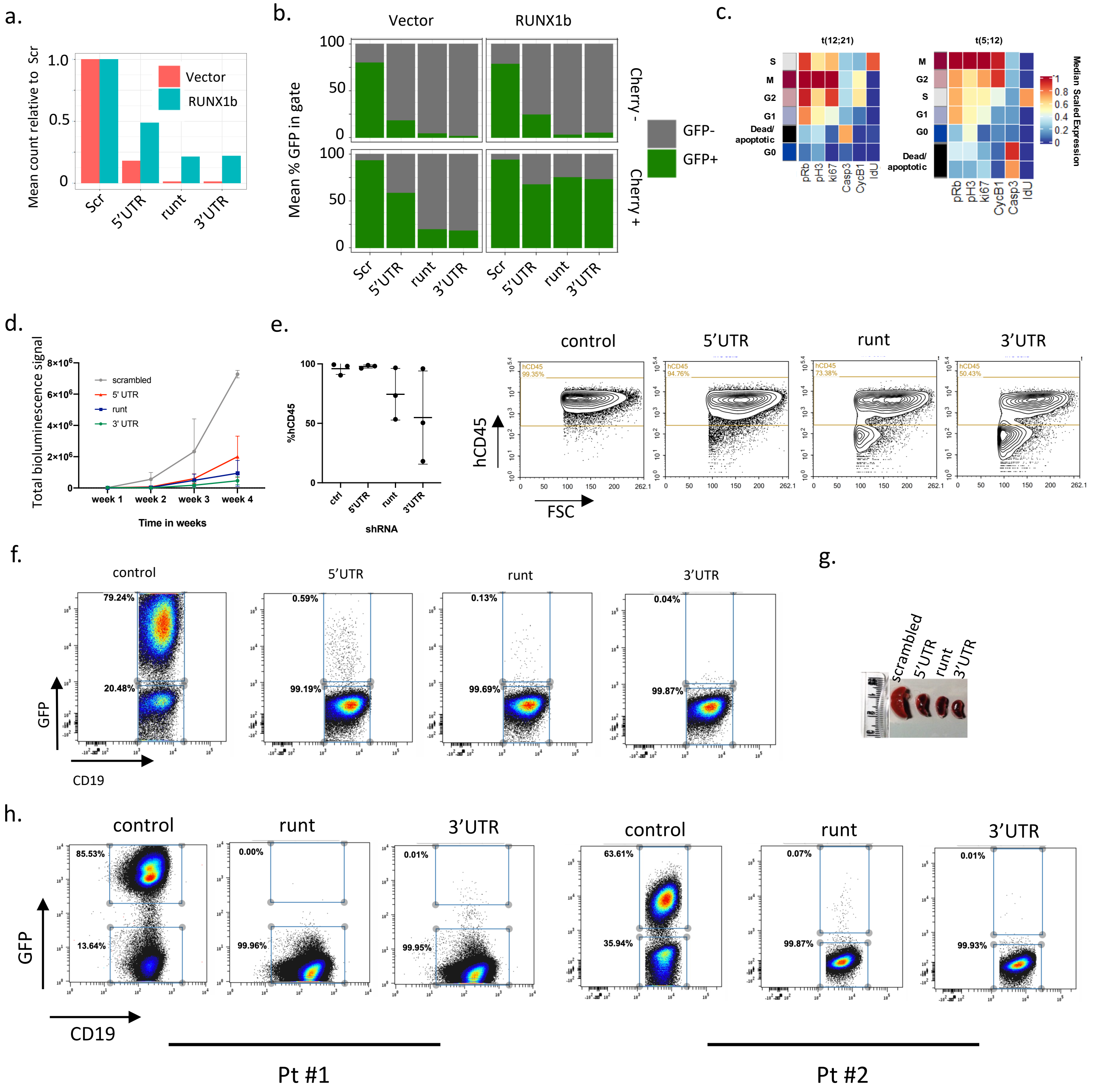

Supplementary Figure 7; related to Figure 7. B-ALL cell lines and primary patient cells are dependent on RUNX1 activity for survival *in vitro* and *in vivo*

(a) Barplot showing the mean number of Cherry+ (Vector/RUNX1b), GFP+ (shRNA) RCH-ACV cells relative to Scr controls (n=2) after 20 days in culture. (b) Barplot showing the proportion of shRNA-expressing (GFP+) cells in the Cherry- and Cherry+ fractions of cells transduced with Vector or RUNX1b after 20 days in culture. (c) Heatmaps showing median scaled expression of the indicated markers in the annotated SOM clusters, corresponding to figure 6B. (d) Summary of bioluminescence imaging of NSG mice engrafted with a 1:1 mix of NALM-6 cells transduced with shRNAs (GFP^+) and stably expressing a luciferase/RFP reporter were mixed 1:1 with non-transduced (GFP^-) over four weeks. Relative bioluminescence signal is estimated as the sum of measured signal of ventral and dorsal positions at each time point (n=3). $p<0.0001$, 2-way Anova test. (e) Left, summary plot showing percentage human CD45 chimerism in NSG mice engrafted with NALM-6 cells (as in d) at end point (4 weeks). Data presented as mean +/- SD, n=3 biologically independent samples; right, representative FACS plots of human CD45 levels at end point. (f) Representative FACS plot of end point (4 weeks) analysis of NSG mice engrafted with NALM-6 cells as in Figure 7c. (g) Representative image of splenic size obtained from NSG mice engrafted with a 1:1 mix of NALM-6 cells transduced with shRNAs (GFP^+) and stably expressing a luciferase/RFP reporter were mixed 1:1 with non-transduced (GFP^-) at week four (end point). (h) Representative FACS plot of endpoint (3 months) analysis of NSG mice competitively engrafted with primary B-ALL cells. Human ($CD19^+$) engraftment and the proportion of shRNA-transduced (GFP^+) cells for two ALL patient samples. Source data are provided as a Source Data file.

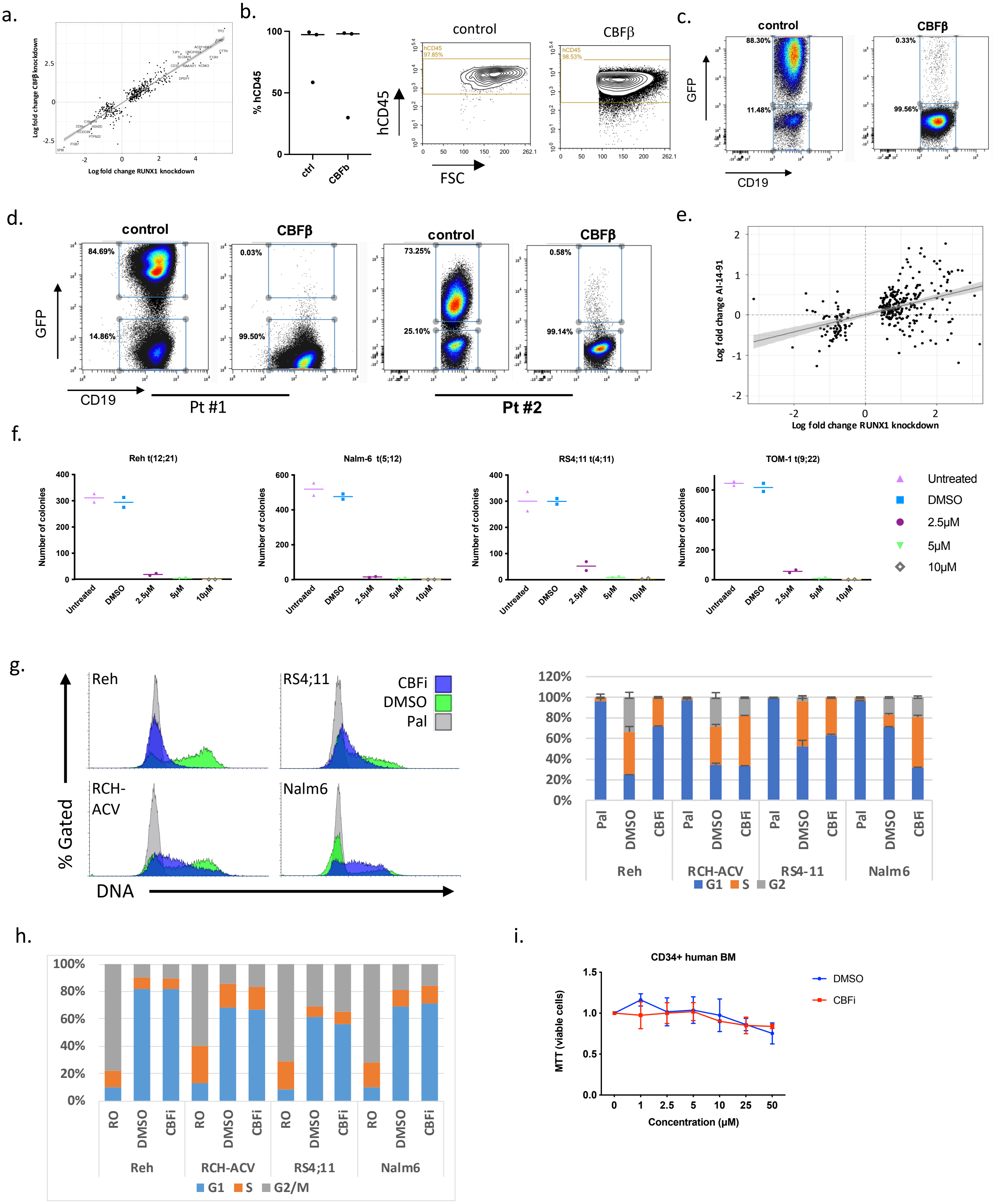
a.
Log fold change CBFβ knockdown
Log fold change RUNX1 knockdown
b.
% hCD45
ctrl
CBFb
control
CBFβ
hCD45
FSC
hCD45
97.85%
hCD45
98.53%
c.
control
CBFβ
GFP
CD19
88.30%
11.48%
0.33%
99.56%
d.
control
CBFβ
control
CBFβ
GFP
CD19
84.69%
14.86%
0.03%
99.50%
73.25%
25.10%
0.58%
99.14%
Pt #1
Pt #2
e.
Log fold change AI-14-91
Log fold change RUNX1 knockdown
f.
Reh t(12;21)
Nalm-6 t(5;12)
RS4;11 t(4;11)
TOM-1 t(9;22)
Number of colonies
Untreated
DMSO
2.5μM
5μM
10μM
g.
Reh
RS4;11
RCH-ACV
Nalm6
CBFi
DMSO
Pal
% Gated
DNA
Pal
DMSO
CBFi
Reh
RCH-ACV
RS4-11
Nalm6
G1
S
G2
h.
RO
DMSO
CBFi
Reh
RCH-ACV
RS4;11
Nalm6
G1
S
G2/M
i.
CD34+ human BM
MTT (viable cells)
Concentration (μM)
DMSO
CBFi

Supplementary Figure 8; related to Figure 8. An allosteric CBFβ inhibitor mimics RUNX1 depletion phenotype and offers a targeted treatment for B-ALL

(a) Dotplot comparing log fold change following RUNX1 knockdown (x-axis) and CBFb knockdown (y-axis) in ALL cell lines. “Core” RUNX1 target genes defined in Figure 5 are shown. (b) Left, summary plot showing percentage human CD45 chimerism in NSG mice engrafted with NALM-6 cells (as in Figure 7d) at end point (4 weeks), n=3 biologically independent samples; right, representative FACS plots of human CD45 levels at end point. (c) Representative FACS plot of endpoint (4 weeks) analysis of NSG mice engrafted with NALM-6 cells as in Figure 7d.(d) Representative FACS plot of endpoint (3 months) analysis of NSG mice competitively engrafted with primary B-ALL cells. Human ($CD19^{+}$) engraftment and the proportion of shRNA-transduced (GFP^{+}) cells for two ALL patient samples with the indicated genotypes. (e) Dotplot comparing log fold change following RUNX1 knockdown (x-) and CBFi (AI-14-91) treatment (y-axis) in ALL cell lines. “Core” RUNX1 target genes defined in Figure 5 are shown. (f) Colony formation assay of B-ALL cell lines treated with increasing concentrations of CBFi (AI-14-91) (n=2), see Methods for more details. (g) Histograms of DNA content (Hoechst 33342, left panel) showing the impact of AI-14-91 (CBFi) on cell cycle re-entry following palbociclib (Pal) wash-out. Following wash-out cells were incubated for a further 18h in the presence of Pal, CBFi or DMSO. Quantification (right panel) of cell cycle distribution based on DNA content (G1=2N, $2N<S<4N$, G2=4N). Error bars indicate standard deviation (n=3). Note that NALM-6 transit through the cell cycle more rapidly, hence a high proportion of control (DMSO) cells have re-entered G1. (h) As in (g) but cells were arrested in G2 with RO3306 (RO). (i) Plot showing sensitivity of $CD34^{+}$ human bone marrow (hBM) cells to AI-14-91 (CBFi). Cells were cultured in the indicated concentrations of CBFi or vehicle control (DMSO) for 48h and MTT assay for cell viability performed. Values were normalised to untreated (0mM, DMSO) cells. Error bars indicate standard deviation (n=3). Source data are provided as a Source Data file.

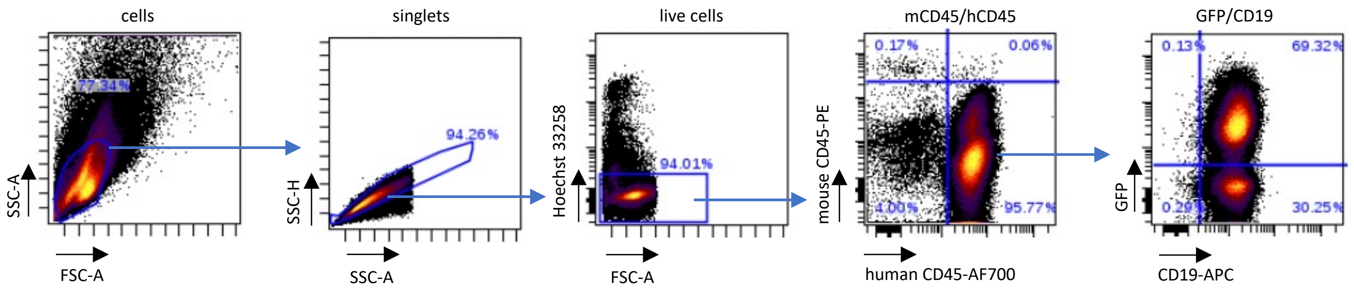

Supplementary Figure 9. Representative gating strategy for figures 7C, 7D, 8C, and supplementary figures 7E, 7F, 7H, 8B, 8C, and 8D.

Cells were gated on forward scatter (FSC) and side scatter (SSC), followed by singlet gating. Live cells (Hoechst 33258 low) were then separated into mouse and human $CD45^+$ cells. The human $CD45^+$ cells were then gated on GFP and human CD19 to facilitate quantification of competitive engraftment.

Isotope-Metal	Antigen/Target	Clone	Dilution
148Nd	CD34	581	1 in 100
147Sm	pHis H2AX	JBW301	1 in 50
159Tb	p21	12D1	1 in 100
89Y	CD45	HI30	1 in 100
165Ho	CD19	HIB19	1 in 100
176Yb	cMyc	9E10	1 in 100
155Gd	CD45RA	HI100	1 in 50
153Eu	CyclinB1	N/A	1 in 100
142Nd	Casp3	D3E9	1 in 100
143Nd	cPARP	F21-852	1 in 100
166Er	pRb	J112-906	1 in 100
175Lu	pHisH3	HTA28	1 in 100
172Yb	Ki67	B56	1 in 200
165Ho	CD127	A019D5	1 in 50

Supplementary Table 1. Mass cytometry antibodies

Cell line	Disease	Cytogenetics	Age	Disease stage
Reh	B-ALL	t(12;21)	15 y.o.	1st relapse
NALM-6	B-ALL	t(5;12)	19 y.o.	relapse
RCH-ACV	B-ALL	t(1;19)	8 y.o.	relapse
RS4;11	B-ALL	t(4;11)	32 y.o.	1st relapse
TOM-1	B-ALL	t(9;22)	54 y.o.	relapse
K562	CML	t(9;22)	52 y.o.	blast crisis

Supplementary Table 2. Cell lines

Patient	Disease type	Risk stratification	Translocation	Age group
Pt#1	B-ALL	high risk	-	childhood
Pt#2	B-ALL	high risk	t(4;11)	childhood

Supplementary Table 3. Patient samples

Fluorochrome	Antigen/Target	Clone	Dilution
PE	mouse CD45	30-F11	1 in 100
AF700	human CD45	HI30	1 in 100
APC	CD19	SJ25C1	1 in 100

Supplementary Table 4. Flow cytometry antibodies

shRNA ID	Sequence (5' to 3')	Region targeted	Vector
EDR12	GCAAGATTCGTGGAAGATAGT	5'UTR	LLX 3.7
EDR19	GGATGTTCCAGATGGCACTCT	runt domain	LLX 3.7
EDR58	GCCTTGAAATACCTGTTTCTT	3' UTR	LLX 3.7
EDCBFb	TGCCACAGGAACCAATCTGTC T	CBFb	LLX 3.7
non-targeting	TCCTAAGGTTAAGTCGCCCT	non-targeting	LLX 3.7
251	GCAACACGTTTGAAATGAAT	ETV6 Exon 3	LLX 3.7
818	TTGTCGTGATAGGTGACCTGGA	ETV6 Exon 5	LLX 3.7

Supplementary Table 5. shRNA sequences
