# Supplementary material for: Regulome analysis in B-acute lymphoblastic leukemia exposes Core Binding Factor addiction as a therapeutic vulnerability
Source: Nat Commun. 2022 Nov 21;13:7124. doi: 10.1038/s41467-022-34653-3 (PMC9678885; doi:10.1038/s41467-022-34653-3)
Supplement: Supplementary file 3 — Reporting Summary [file 41467_2022_34653_MOESM3_ESM.pdf]

nature portfolio

Corresponding author(s): Professor Tariq Enver

Last updated by author(s): Jul 6, 2022

Reporting Summary

Nature Portfolio wishes to improve the reproducibility of the work that we publish. This form provides structure for consistency and transparency in reporting. For further information on Nature Portfolio policies, see our Editorial Policies and the Editorial Policy Checklist.

Statistics

For all statistical analyses, confirm that the following items are present in the figure legend, table legend, main text, or Methods section.

n/a	Confirmed	
[ ]	[x]	The exact sample size (*n*) for each experimental group/condition, given as a discrete number and unit of measurement
[x]	[ ]	A statement on whether measurements were taken from distinct samples or whether the same sample was measured repeatedly
[ ]	[x]	The statistical test(s) used AND whether they are one- or two-sided<br>*Only common tests should be described solely by name; describe more complex techniques in the Methods section.*
[x]	[ ]	A description of all covariates tested
[ ]	[x]	A description of any assumptions or corrections, such as tests of normality and adjustment for multiple comparisons
[ ]	[x]	A full description of the statistical parameters including central tendency (e.g. means) or other basic estimates (e.g. regression coefficient) AND variation (e.g. standard deviation) or associated estimates of uncertainty (e.g. confidence intervals)
[ ]	[x]	For null hypothesis testing, the test statistic (e.g. *F*, *t*, *r*) with confidence intervals, effect sizes, degrees of freedom and *P* value noted<br>*Give P values as exact values whenever suitable.*
[x]	[ ]	For Bayesian analysis, information on the choice of priors and Markov chain Monte Carlo settings
[x]	[ ]	For hierarchical and complex designs, identification of the appropriate level for tests and full reporting of outcomes
[ ]	[x]	Estimates of effect sizes (e.g. Cohen's *d*, Pearson's *r*), indicating how they were calculated

Our web collection on statistics for biologists contains articles on many of the points above.

Software and code

Policy information about availability of computer code

Data collection

No software used in data collection

Data analysis

All software used are open source and listed in our methods section. ChIP and RNA-seq data were subjected to QC, mapped and counts matrices generated using Nextflow pipelines. ChIP: https://github.com/nf-core/chipseq. RNA: https://github.com/nf-core/rnaseq. Further analysis was carried out using published, open source packages for R. RStudio Version 1.4.1106, R version 4.0.5, Bioconductor 3.12.

The following were employed in data analysis:

ChIP-seq:
MACS (Zhang et al., 2008)
NGSPlot (Shen, 2014)
Rsubread::featureCounts (Liao et al, 2019)
Integrative Genomics Viewer (Robinson et al, 2011)
MEME-ChIP (Machanick et al, 2011)
ChIPpeakAnno (Zhu et al, 2010)
deepTools2 (Fidel et al, 2016)
DiffBind (Stark and Brown, 2011)

RNAseq:
Analyses were performed within the R statistical computing framework, version 4.0.2 using packages from BioConductor version 3.11 (https://

Bioconductor.org)
DEseq2 (Love et al, 2014)
ggplot2 (Wickham, 2016)
pheatmap (Kolde, 2019)
clusterProfiler (Yu et al, 2012)

Mass cytometry:
Analyses were performed within the R statistical computing framework, version 4.0.2 using packages from BioConductor version 3.11 (https://Bioconductor.org)
CATALYST (Crowell et al, 2020)
Cytobank (https://mrc.cytobank.org/)

For manuscripts utilizing custom algorithms or software that are central to the research but not yet described in published literature, software must be made available to editors and reviewers. We strongly encourage code deposition in a community repository (e.g. GitHub). See the Nature Portfolio guidelines for submitting code & software for further information.

Data

Policy information about availability of data

All manuscripts must include a data availability statement. This statement should provide the following information, where applicable:

- Accession codes, unique identifiers, or web links for publicly available datasets
- A description of any restrictions on data availability
- For clinical datasets or third party data, please ensure that the statement adheres to our policy

The datasets generated during and/or analysed during the current study are available in the ArrayExpress repository.
https://www.ebi.ac.uk/biostudies/arrayexpress/studies/E-MTAB-10308
https://www.ebi.ac.uk/biostudies/arrayexpress/studies/E-MTAB-6382
https://www.ebi.ac.uk/biostudies/arrayexpress/studies/E-MTAB-10329
https://www.ebi.ac.uk/biostudies/arrayexpress/studies/E-MTAB-10312
https://www.ebi.ac.uk/biostudies/arrayexpress/studies/E-MTAB-12207
https://www.ebi.ac.uk/biostudies/arrayexpress/studies/E-MTAB-12208
https://www.ebi.ac.uk/biostudies/arrayexpress/studies/E-MTAB-12209

Human research participants

Policy information about studies involving human research participants and Sex and Gender in Research.

Reporting on sex and gender	*Use the terms sex (biological attribute) and gender (shaped by social and cultural circumstances) carefully in order to avoid confusing both terms. Indicate if findings apply to only one sex or gender; describe whether sex and gender were considered in study design whether sex and/or gender was determined based on self-reporting or assigned and methods used. Provide in the source data disaggregated sex and gender data where this information has been collected, and consent has been obtained for sharing of individual-level data; provide overall numbers in this Reporting Summary. Please state if this information has not been collected. Report sex- and gender-based analyses where performed, justify reasons for lack of sex- and gender-based analysis.*
Population characteristics	*Describe the covariate-relevant population characteristics of the human research participants (e.g. age, genotypic information, past and current diagnosis and treatment categories). If you filled out the behavioural & social sciences study design questions and have nothing to add here, write "See above."*
Recruitment	Patient bone marrow samples were obtained from Great Ormond Street Hospital for Children diagnostic archives. Informed consent was obtained from all participants.
Ethics oversight	National Research Ethics Service Committee London Brent, reference 16/LO/0960

Note that full information on the approval of the study protocol must also be provided in the manuscript.

Field-specific reporting

Please select the one below that is the best fit for your research. If you are not sure, read the appropriate sections before making your selection.

☒ Life sciences ☐ Behavioural & social sciences ☐ Ecological, evolutionary & environmental sciences

For a reference copy of the document with all sections, see nature.com/documents/nr-reporting-summary-flat.pdf

Life sciences study design

All studies must disclose on these points even when the disclosure is negative.

Sample size	For animal experiments the experimental design assistant (https://eda.nc3rs.org.uk/) shows that 3 animals/groups are sufficient to detect differences in engraftment >= 50%.
Data exclusions	All sequencing data was assessed to detect sequencing failures using FASTQC and lower quality reads were filtered or trimmed using TrimGalore (https://github.com/FelixKrueger/TrimGalore). Outlier samples containing low sequencing coverage or high duplication rates were discarded.
Replication	Where possible a minimum of three biological replicates were performed. For knock-down experiments multiple shRNAs targeting the same transcript were used. For induced pluripotent stem cell experiments isogenic parental, engineered knock-in (x2) and CRE-reverted cell lines were used.<br><br>For mouse experiments, 3 mice per group were analysed
Randomization	For in vivo experiments mice were randomly assigned to experimental or control groups.
Blinding	Blinding was not possible as most experiments were carried out by a single researcher. Primary readout of animal experiments was engraftment measured by flow cytometry, with consistent gating across control and experimental samples ensuring objectivity in analysis.

Reporting for specific materials, systems and methods

We require information from authors about some types of materials, experimental systems and methods used in many studies. Here, indicate whether each material, system or method listed is relevant to your study. If you are not sure if a list item applies to your research, read the appropriate section before selecting a response.

Materials & experimental systems

n/a	Involved in the study	
[ ]	[x]	Antibodies
[ ]	[x]	Eukaryotic cell lines
[x]	[ ]	Palaeontology and archaeology
[ ]	[x]	Animals and other organisms
[x]	[ ]	Clinical data
[x]	[ ]	Dual use research of concern

Methods

n/a	Involved in the study	
[ ]	[x]	ChIP-seq
[ ]	[x]	Flow cytometry
[x]	[ ]	MRI-based neuroimaging

Antibodies

Antibodies used	RUNX1 (Abcam, ab23980), ETV6: (Sigma, HPA000264), CBF (Abcam, ab33516), GAPDH (14C10, Cell Signaling #2128), V5 (Abcam, ab9116), IgG (Abcam, ab171870), Anti-Histone H3 (acetyl K27) antibody - ChIP Grade (ab4729)<br><br>FACS antibodies - anti mouse CD45-PE (Biolegend), anti human CD45-AF700, CD19-APC (Biolegend)<br><br>Mass cytometry antibodies (148Nd CD34, 147Sm pHis H2AX, 159Tb p21, 89Y CD45, 165Ho CD19, 176Yb cMyc, 155Gd CD45RA, 153Eu CyclinB1, 142Nd Casp3, 143Nd cPARP, 166Er pRb, 175Lu pHisH3, 172Yb Ki67, 165Ho CD127, all Fluidigm)
Validation	Antibodies were validated by western blot using negative controls (non-expressing cell lines (ETV6)), positive controls (forced expression (V5-ETV6-RUNX1, FLAG-RUNX1b) and knock-downs (RUNX1/ETV6-RUNX1/CBFb).<br><br>FACS antibodies were titrated on human leukaemic cells prior to use.<br><br>Mass cytometry antibodies were titrated on human cord blood and leukaemic cells prior to usage. Cell cycle antibodies were validated through the use of CDK inhibitors.

Eukaryotic cell lines

Policy information about cell lines and Sex and Gender in Research

Cell line source(s)	Reh, NALM-6, RCH-ACV, RS4;11 and TOM-1 were purchased from DSMZ. K562 cells were a kind gift from Prof Asim Khwaja, UCL Cancer Institute, Department of Hematology, London, UK. 293T cells were purchased from ATCC. IPSCs (MIFF3) were a gift from Prof Peter Andrews, University of Sheffield.
Authentication	IPSC were authenticated by southern blot and PCR (for engineered ETV6-RUNX1) and subjected to karyotyping (Boiers et al,

	2018). For Reh t(12;21), NALM-6, RCH-ACV and TOM-1 QPCR was performed using Taqman probes or SYBR primers against the fusion genes. K562 and 293T cells were not validated.
Mycoplasma contamination	Not tested
Commonly misidentified lines (See ICLAC register)	N/A

Animals and other research organisms

Policy information about studies involving animals; ARRIVE guidelines recommended for reporting animal research, and Sex and Gender in Research

Laboratory animals	NOD-scid IL2Rgnull (NSG) mice were used at 8-12 weeks old
Wild animals	The study does not involve wild animals
Reporting on sex	Only female animals were used.
Field-collected samples	The study does not involve samples collected from the field
Ethics oversight	All animal experiments were performed in strict accordance with the United Kingdom Home Office regulations. All study protocols used are under done as described in the Enver Project Licence PFEC1FA8A.

Note that full information on the approval of the study protocol must also be provided in the manuscript.

ChIP-seq

Data deposition

☒ Confirm that both raw and final processed data have been deposited in a public database such as GEO.

☐ Confirm that you have deposited or provided access to graph files (e.g. BED files) for the called peaks.

Data access links
May remain private before publication.

https://www.ebi.ac.uk/biostudies/arrayexpress/studies/E-MTAB-10312
https://www.ebi.ac.uk/biostudies/arrayexpress/studies/E-MTAB-12207
https://www.ebi.ac.uk/biostudies/arrayexpress/studies/E-MTAB-12208
https://www.ebi.ac.uk/biostudies/arrayexpress/studies/E-MTAB-12209

Files in database submission

E-MTAB-10312:
Nalm6_ETV6-RUNX1_DnaseHS ftp://ftp.sra.ebi.ac.uk/vol1/fastq/ERR564/004/ERR5643704/ERR5643704.fastq.gz
Nalm6_ETV6-RUNX1-dHLH _DnaseHS ftp://ftp.sra.ebi.ac.uk/vol1/fastq/ERR564/005/ERR5643705/ERR5643705.fastq.gz
Nalm6_ETV6-RUNX1-R139G_DnaseHS ftp://ftp.sra.ebi.ac.uk/vol1/fastq/ERR564/006/ERR5643706/ERR5643706.fastq.gz
Reh_DnaseHS_A ftp://ftp.sra.ebi.ac.uk/vol1/fastq/ERR564/007/ERR5643707/ERR5643707.fastq.gz
Reh_DnaseHS_B ftp://ftp.sra.ebi.ac.uk/vol1/fastq/ERR564/008/ERR5643708/ERR5643708.fastq.gz
Nalm6_CSI_DnaseHS ftp://ftp.sra.ebi.ac.uk/vol1/fastq/ERR564/009/ERR5643709/ERR5643709.fastq.gz
Patient1_HPA ftp://ftp.sra.ebi.ac.uk/vol1/fastq/ERR564/000/ERR5643710/ERR5643710.fastq.gz
Patient2_HPA ftp://ftp.sra.ebi.ac.uk/vol1/fastq/ERR564/001/ERR5643711/ERR5643711.fastq.gz
Patient3_HPA ftp://ftp.sra.ebi.ac.uk/vol1/fastq/ERR564/002/ERR5643712/ERR5643712.fastq.gz
Reh_CBFi_HPA ftp://ftp.sra.ebi.ac.uk/vol1/fastq/ERR564/003/ERR5643713/ERR5643713.fastq.gz
Reh_DMSO_HPA ftp://ftp.sra.ebi.ac.uk/vol1/fastq/ERR564/004/ERR5643714/ERR5643714.fastq.gz
Reh_HPA ftp://ftp.sra.ebi.ac.uk/vol1/fastq/ERR564/005/ERR5643715/ERR5643715.fastq.gz
Reh_CBFi_IgG ftp://ftp.sra.ebi.ac.uk/vol1/fastq/ERR564/006/ERR5643716/ERR5643716.fastq.gz
Reh_DMSO_IgG ftp://ftp.sra.ebi.ac.uk/vol1/fastq/ERR564/007/ERR5643717/ERR5643717.fastq.gz
Patient1_PAS ftp://ftp.sra.ebi.ac.uk/vol1/fastq/ERR564/008/ERR5643718/ERR5643718.fastq.gz
Patient2_PAS ftp://ftp.sra.ebi.ac.uk/vol1/fastq/ERR564/009/ERR5643719/ERR5643719.fastq.gz
Patient3_PAS ftp://ftp.sra.ebi.ac.uk/vol1/fastq/ERR564/000/ERR5643720/ERR5643720.fastq.gz
Reh_PAS ftp://ftp.sra.ebi.ac.uk/vol1/fastq/ERR564/001/ERR5643721/ERR5643721.fastq.gz
Nalm6_RUNX1 ftp://ftp.sra.ebi.ac.uk/vol1/fastq/ERR564/002/ERR5643722/ERR5643722.fastq.gz
RCH-ACV_RUNX1 ftp://ftp.sra.ebi.ac.uk/vol1/fastq/ERR564/003/ERR5643723/ERR5643723.fastq.gz
Reh_CBFi_RUNX1 ftp://ftp.sra.ebi.ac.uk/vol1/fastq/ERR564/004/ERR5643724/ERR5643724.fastq.gz
Reh_DMSO_RUNX1 ftp://ftp.sra.ebi.ac.uk/vol1/fastq/ERR564/005/ERR5643725/ERR5643725.fastq.gz
Reh_RUNX1_B ftp://ftp.sra.ebi.ac.uk/vol1/fastq/ERR564/006/ERR5643726/ERR5643726.fastq.gz
Nalm6_CSI_V5 ftp://ftp.sra.ebi.ac.uk/vol1/fastq/ERR564/007/ERR5643727/ERR5643727.fastq.gz
Reh_CSI_V5 ftp://ftp.sra.ebi.ac.uk/vol1/fastq/ERR564/008/ERR5643728/ERR5643728.fastq.gz
Nalm6_ETV6-RUNX1_V5_B ftp://ftp.sra.ebi.ac.uk/vol1/fastq/ERR564/009/ERR5643729/ERR5643729.fastq.gz
Nalm6_ETV6-RUNX1-dHLH _V5_B ftp://ftp.sra.ebi.ac.uk/vol1/fastq/ERR564/000/ERR5643730/ERR5643730.fastq.gz
Nalm6_ETV6-RUNX1-R139G_V5 ftp://ftp.sra.ebi.ac.uk/vol1/fastq/ERR564/001/ERR5643731/ERR5643731.fastq.gz

E-MTAB-12207:

C_N6_csi_K27ac.qc.adapt.fastq.gz
C_N6_csi_K27ac_b.qc.adapt.fastq.gz
C_N6_TA_K27ac.qc.adapt.fastq.gz
C_N6_TA_K27ac_b.qc.adapt.fastq.gz
C_N6_TAdH_K27ac.qc.adapt.fastq.gz
C_N6_TAdH_K27ac_b.qc.adapt.fastq.gz
C_N6_TARG_K27ac.qc.adapt.fastq.gz
C_N6_TARG_K27ac_b.qc.adapt.fastq.gz

E-MTAB-12208:
A042-ED-ChIP-NextSeq_R1.fastq.gz
A042-ED-ChIP-NextSeq_R2.fastq.gz
A033-ED-ChIP-NextSeq_R1.fastq.gz
A033-ED-ChIP-NextSeq_R2.fastq.gz
A031-ED-ChIP-NextSeq_R1.fastq.gz
A031-ED-ChIP-NextSeq_R2.fastq.gz
A036-ED-ChIP-NextSeq_R2.fastq.gz
A036-ED-ChIP-NextSeq_R1.fastq.gz
A034-ED-ChIP-NextSeq_R1.fastq.gz
A034-ED-ChIP-NextSeq_R2.fastq.gz
A026-ED-ChIP-NextSeq_R2.fastq.gz
A026-ED-ChIP-NextSeq_R1.fastq.gz
A029-ED-ChIP-NextSeq_R1.fastq.gz
A029-ED-ChIP-NextSeq_R2.fastq.gz

E-MTAB-12209:
A017-ED-ChIPrev1-NS_R1.fastq.gz
A017-ED-ChIPrev2-NS_R1.fastq.gz
A047-ED-ChIPrev2-NS_R1.fastq.gz
A047-ED-ChIPrev1-NS_R1.fastq.gz
A048-ED-ChIPrev2-NS_R1.fastq.gz
A048-ED-ChIPrev1-NS_R1.fastq.gz
A027-ED-ChIPrev1-NS_R1.fastq.gz
A027-ED-ChIPrev2-NS_R1.fastq.gz
A045-ED-ChIPrev1-NS_R1.fastq.gz
A045-ED-ChIPrev2-NS_R1.fastq.gz
A046-ED-ChIPrev2-NS_R1.fastq.gz
A046-ED-ChIPrev1-NS_R1.fastq.gz
A019-ED-ChIPrev2-NS_R1.fastq.gz
A019-ED-ChIPrev1-NS_R1.fastq.gz
A020-ED-ChIPrev1-NS_R1.fastq.gz
A020-ED-ChIPrev2-NS_R1.fastq.gz
A021-ED-ChIPrev1-NS_R1.fastq.gz
A021-ED-ChIPrev2-NS_R1.fastq.gz
A015-ED-ChIPrev2-NS_R1.fastq.gz
A015-ED-ChIPrev1-NS_R1.fastq.gz
A016-ED-ChIPrev1-NS_R1.fastq.gz
A016-ED-ChIPrev2-NS_R1.fastq.gz
A018-ED-ChIPrev1-NS_R1.fastq.gz
A018-ED-ChIPrev2-NS_R1.fastq.gz

Genome browser session
(e.g. UCSC)

https://genome-euro.ucsc.edu/s/BALL_project/BALL_ChIP_data

Methodology

Replicates

Native ETV6-RUNX1 ChIP was performed with two independent antibodies in three patient samples and one cell line. A high degree of correlation was observed between antibodies and samples.

RUNX1 ChIP was performed in three cell lines. A high degree of correlation was observed between cell lines. Overlapping peaks were identified to define those shared across B-ALL sub-types

Sequencing depth

20-40 million reads per sample were obtained

Antibodies

ETV6 antibodies (for ETV6-RUNX1 ChIP): "HPA", Sigma HPA000264; "PAS" a gift from OA Bernard
RUNX1: Abcam Ab 23980
V5: Abcam 9116
Anti-Histone H3 (acetyl K27) antibody - ChIP Grade (ab4729)

Peak calling parameters

Peaks were called by MACS with the following parameters:

effective genome size = 2.70e+09
band width = 300
model fold = [5, 50]
qvalue cutoff = 1.00e-02

Control files were IgG ChIP or V5 ChIP performed in vector control samples.

Data quality	All ChIP samples were examined visually for robust peaks. Furthermore, only peaks overlapping DNaseHS sites were considered for further analyses.
Software	nfcore pipeline https://github.com/nf-core/chipseq<br>MACS (Zhang et al., 2008)<br>NGSPlot (Shen, 2014)<br>Rsubread::featureCounts (Liao et al, 2019)<br>Integrative Genomics Viewer (Robinson et al, 2011)<br>MEME-ChIP (Machanick et al, 2011)<br>ChIPpeakAnno (Zhu et al, 2010)<br>deepTools2 (Fidel et al, 2016)<br>DiffBind (Stark and Brown, 2011)

Flow Cytometry

Plots

Confirm that:

- [x] The axis labels state the marker and fluorochrome used (e.g. CD4-FITC).
- [x] The axis scales are clearly visible. Include numbers along axes only for bottom left plot of group (a 'group' is an analysis of identical markers).
- [x] All plots are contour plots with outliers or pseudocolor plots.
- [x] A numerical value for number of cells or percentage (with statistics) is provided.

Methodology

Sample preparation	or flow cytometry analysis or sorting, cell lines or primary patient cells were centrifuged at 300rcf for 5min and washed in 1ml PBS/2%FBS. Staining was done in 100ml volume. A master mix with appropriate antibodies was prepared and added to each sample. Unstained and single stain controls were included for each experiment and Hoechst 33258 (SigmaAldrich) was used as a viability dye.<br>Prior to staining cells obtained from animals cells were lysed with 1ml of Red Blood Cell (RBC) lysis buffer (SigmaAldrich) and incubated for 5-10 min at room temperature.
Instrument	Gallios (Beckman Coulter) was used for FACS analysis. ARIA III (BD) was used for sorting
Software	Kaluza Software (Beckman Coulter) and Cytobank platform (Beckman Coulter)
Cell population abundance	FACS-sorting of cells for RNA-Seq or engraftment experiments was done on "purity mask" and stringent gating of the positive population (significant gap between positive and negative gates). Purity of sorted cells was checked by running the sorted sample and confirming that >90% of sorted cells were falling into the gate of interest.
Gating strategy	All negative and positive gates were drawn based on unstained control cells, single stained fluorescent beads and FMO (fluorescence minus 1) controls where necessary. 1) Cells were gated on FSC/SSC to exclude debris. 2) FSC-H vs FSC-A was then used to select singlets and exclude doublet populations. Hoechst 33258 (SigmaAldrich) was used as a viability marker and Hoechst33258-negative cells were gated as live. For assessing level of patient material engraftment in NSG mice samples were stained with anti mouse CD45 (PE), anti human CD45 (AF700) and CD19 (APC). Live Hoechst33258-negative cells were gated for mouse CD45 and human CD45 to assess proportion of human blood cells. Human CD45-positive cells were also gated on CD19 as an additional marker for B-lymphoid cells. For competitve engraftement experiments live CD45+CD19+ cells were gated on GFP (FITC) to assess transduction efficiency and level of remaining shRNA-transduced (GFP-positive) human cells.

- [x] Tick this box to confirm that a figure exemplifying the gating strategy is provided in the Supplementary Information.
